# Diagnostic Capability of Capsule Endoscopy in Small Bowel Diseases

**DOI:** 10.4021/gr2009.03.1281

**Published:** 2009-03-20

**Authors:** Filiz Akyuz, Zeynel Mungan

**Affiliations:** a Department of Gastroenterohepatology, Istanbul Medical Faculty, Istanbul University, Turkey

**Keywords:** Capsule endoscopy, Indication, Small bowel

## Abstract

Capsule Endoscopy (CE) is a recently developed noninvasive technique for imaging of small bowel pathologies. It is a swallowable wireless mini-camera for getting images of the gastrointestinal (GI) mucosa. General indications of CE are obscure bleeding, iron deficiency anemia, Crohn disease, abdominal pain, polyposis coli, celiac disease and small bowel tumors. Obstruction must be excluded with small bowel radiography before using CE. Bowel preparation can be recommended for good visualization. The main indication is obscure GI bleeding. Even though useful for the other indications in selected cases, large polypoid lesions may be missed. Diagnostic capability of CE and double balloon enteroscopy (DBE) are similar and CE is a good complemantary method for DBE.

Wireless Capsule Endoscopy (WCE) is a recently developed noninvasive technique for imaging of small bowel pathologies. In 1995, Gavriel Idan first presented the idea of WCE to Applitec Ltd. It was improved by “Given Imaging Research and Development” in Israel in 1999. Later it was approved in Western countries in 2001. WCE is a swallowable wireless mini-camera for getting images of the gastrointestinal (GI) mucosa [1-3]. The system has an external receiving antenna with attached portable hard disc drive (data recorder) and customized PC Workstation. Data recorder records the views for 8 hours. During this period patient can continue working. At the end of this period, data is downloaded to the computer, and doctor analyses the results, generally, this takes 1 hour.

Widely accepted indications of WCE are as follows [1-3], obscure bleeding; iron deficiency anemia; crohn disease; abdominal pain; polyposis coli; celiac disease; Small bowel tumors.

In some selected patients, it can be used to monitor the small bowel in renal/bone marrow transplanted patients, in management of graft versus-host disease, monitoring deleterious effects of drugs (Nonsteroid anti-inflammatory drugs), after small bowel transplantation and in chronic refractory pouchitis [4-6].

Although capsule may retain safely for a long time in a small bowel [7], obstruction must be excluded before CE with small bowel radiography or computerized tomography (CT) with oral contrast when indicated. Retained capsule is the indication for surgery, but nowadays it can be taken out by double balloon enteroscopy (DBE) (Fig. 1a-c). Retained capsule also can be a positive sign in the malign obstruction for surgery. All these findings show that retained capsule which is the main complication of CE, is not accepted as a complication nowadays. In our experience, to exclude obstructive lesions, we prefer CT enteroclysis. Small bowel radiography can miss obstruction. In our cases, capsule retained in 4 out of 86 patients, one was out after one week, two underwent operation (one for multiple strictures, one for adenocancer), and one was taken out by DBE. All of these patients had small bowel radiogram which was reported normal. We performed CE, in spite of the high risk retention in only one patient who had multiple strictures. After CE retention, we should wait at least two weeks. CT enteroclysis is not only to show obstructive lesions but also to help exclude other pathologies in abdomen which can cause external impression, such as extraintestinal tumors, lympadenopathy, lymphoma.

**Figure 1:**
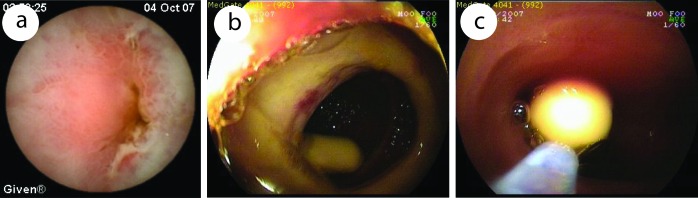
Capsule endoscopic view of a 31 years old patient diagnosed of diaphragm diseased caused by NSAID. (a), capsule retained in narrowed ulcer area. (b, c), taking out of retained capsule by DBE.

Contraindications of WCE are as follows. The absolute contraindications are Stricture, obstruction, fistulas, widespread Crohn disease**,** swallowing problems, pseudoobstruction, Motility problems. The relative contraindications are pregnancy, long term use of NSAIDs, large diverticula, Zenker diverticula, gastroparesia, history of surgical operations.

Bowel preparation is important for the success of procedure. Good preparation is also important for good visualization. The diagnostic yield of CE depends on the quality of visualization of the small-bowel wall and complete passage through the small bowel.

Although one multicenter randomized controlled trial including 129 patients did not support the recommendation of small-bowel preparation with oral NaP for CE exploration in patients with occult gastrointestinal bleeding [8]. In most studies, bowel preparation offers better visualization than overnight fasting alone and is associated with fewer disturbances due to intraluminal turbid fluid. Because of this, bowel preparation is recommended before capsule endoscopy [9-12].

Currently, obscure GI bleeding is the most common indication of CE. The diagnostic yield of CE has been reported to be 38% to 93% and most common lesions in patients with obscure bleeding are as follows [13-16], angioectasia, tumor, varices, diverticula and ulcer.

CE has a high diagnostic yield (91.9%) in patients with overt active bleeding and the most common findings were in order of angiodysplasia, ulcer, polyp, tumor [17]. Hartmann et al [18] showed that CE (74.4%) has same diagnostic value with intraoperative enteroscopy (72.3%). They evaluated forty-seven consecutive patients with obscure GI bleeding (11 with ongoing overt bleeding, 24 with previous overt bleeding, and 12 with obscure-occult bleeding) from German gastroenterology centers. In comparison to intraoperative enteroscopy (IOE), the sensitivity, specificity, positive and negative predictive values of capsule endoscopy were 95%, 75%, 95% and 86%, respectively. Diagnostic yield was 100% in overt bleeding by CE.

The suspected blood indicator (SBI) feature of CE was developed for rapid screening of intestinal lesions with bleeding potential. However, it is not useful in routine practice. Buscaglia et al [19] showed that the performance characteristics of the currently available SBI feature in CE are suboptimal and insufficient to screen for lesions with bleeding potential. The sensitivity and positive predictive value were low for actively bleeding lesions (58.3% and 70%, respectively). The sensitivity was 58.3% and 41.3% for obscure GI bleeding and anemia, respectively.

CE is also useful for the long-term outcome of obscure GI bleeding. Lai et al [20] followed 49 patients for a median of 19 months (12-31 months), 63.3% of these patients were capsule positive (possible bleeding lesions were detected) and 36.7% were capsule negative. Rebleeding rate was 32.7% in the first year [17]. They showed that the patients with obscure GI bleeding and negative CE had a very low rebleeding rate. CE is accepted as the first line technique in patients with obscure bleeding in American Gastroenterological Association algorithm (Fig. 2) [21].

**Figure 2:**
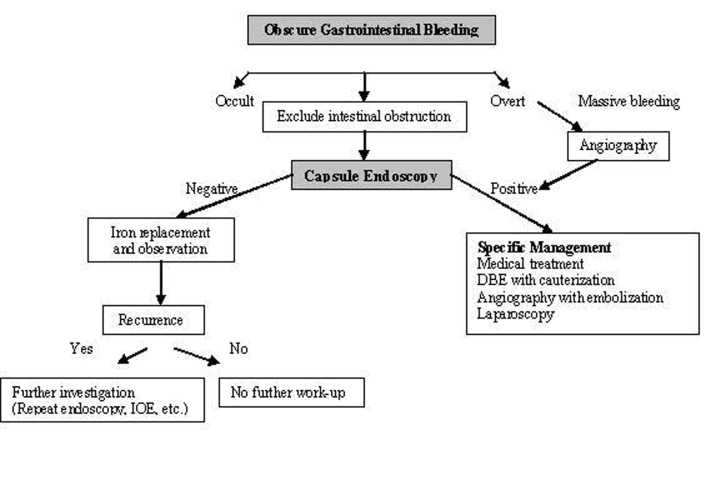
Algorithm for the diagnosis and management of obscure GI bleeding (Adapted from AGA algorithm) [21].

Clinical use of CE in inflammatory bowel disease is limited. Diagnosis of Crohn disease (CD) is based on clinical, endoscopic, radiologic, histologic and biochemical tests. Sometimes diagnosis can be a problem and CE may be useful for diagnosis in non-stricturing small bowel CD. Capsule retention rate is approximately 1.5% in patients with suspected CD and 5-13% in patients with diagnosed CD. Although Triester et al [22] showed that CE is superior to alternative imaging methods for diagnosing non-stricturing small bowel CD in their meta-analysis of 16 studies, it should be remembered that CE shows only the presence of ulcers. The other causes, such as NSAIDs (Fig. 3) and infections, must be excluded before making a diagnosis of Crohn disease. However, in clinical practice, radiographic studies should be performed before CE to exclude obstruction. Therefore, very few patients with CD need CE for diagnosis. In the follow-up period, CE may also be useful in CD patients. It is an effective, safe and well-tolerated method for detecting lesions after surgery (recurrence of CD). It may be helpful for the management of treatment [23]. CE should be considered in ulcerative colitis patients with atypical clinical features [24].

**Figure 3:**
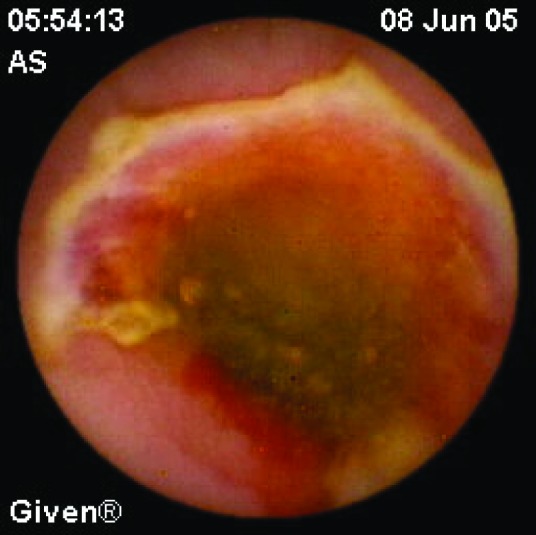
A 61 years old man diagnosed with obscure GI bleeding caused by chronic use of NSAID, showing diaphragm ulcer.

CE has a low yield for evaluation of abdominal pain or diarrhea and cannot be recommended as a first-line test without further studies in patients with only symptom of chronic abdominal pain [25]. May et al [26] evaluated 50 patients with chronic abdominal pain and “plus” symptoms (weight loss, inflammation markers, anemia, or suspected mid-GI bleeding, diarrhea) in a prospective multicenter trial. The additional symptoms or signs of inflammation were associated with the highest diagnostic yield (odds ratio 3.2). They concluded that strict patient selection on the basis of additional symptoms or signs is the key for increasing the yield of CE in patients with chronic abdominal pain and inflammation.

GI polyposis syndromes are defined as the presence of multiple polypoid lesions in the GI system. Most of these syndromes are inherited and associated with increased risk of cancer. Endoscopic and intraoperative resection of polyps is recommended. CE is an alternative imaging technique for surveillance in patients with hereditary polyposis. CE has high diagnostic yield for small polyps (< 15 mm), but larger polyps can be missed [27].

Serology and biopsy is important for the diagnosis of celiac disease and CE is unlikely to replace completely duodenal biopsies, but it produces high quality images of small intestine. It may be useful in assessing patients with celiac disease, and may show entire small bowel and complications of celiac disease (ulcerative jejunoileitis and lymphoma) [28]. Biopsy is not ideal as the gold standard for diagnosis of celiac disease. Mucosal lesions may be patchy thus missed by biopsy and gastroscopy. Rondonotti et al [29] evaluated the potential of videocapsule endoscopy in assessing the severity and extent of mucosal changes in patients with suspected celiac disease. They reported that celiac disease has a high sensitivity (87.5%) and specificity (90.9%) for the detection of villous atrophy in patients with suspected celiac disease. CE may be an option to recognize villous atrophy in patients with a positive endomysial antibody test who are unwilling, or unable to have a gastroscopy [30].

Small bowel malignencies are uncommon and account for less than 6% of all the GI neoplasms [31]. Detection rate has been limited by the inability to endoscopically examine the entire small intestine. Now, it is able to diagnose small bowel malignancies by CE and double balloon enteroscopy (DBE). Most common lesions of small bowel detected by DBE are adenocarcinoma, gastrointestinal stromal tumor, neuroendocrine tumor, lymphoma, cavernous hemangioma, lipoma and hamartoma [32]. Percentages of these lesions are variable in different series.

CE has some potential limitations when used to diagnose small bowel tumors, these limitations are inability to provide histological confirmation, differantiation between malign and benign lesion, missed polypoid lesions [33, 34].

Ross et al [32] evaluated 183 patients presented obscure GI bleeding by DBE. A small bowel mass lesion was detected in 18 patients, 15 of them had prior CE, and mass lesion was identified in 5 patients by CE, but it failed to identify all four cases of primary SB adenocarcinoma. Pasha et al [35] reported a meta-analysis of 11 studies that compares CE and DBE. Summary of the study are shown in figure 4. They found similar diagnostic capability for both**.**

**Figure 4:**
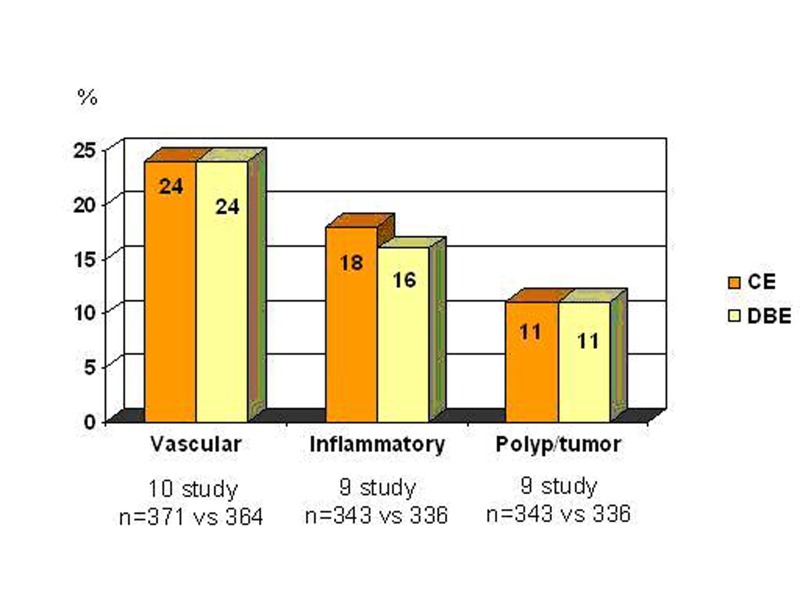
Comparison of CE and DBE (with kindly permission of author’s) [35]

In another study, it was found that an initial DBE is a cost effective approach for patients with obscure bleeding. But they concluded that capsule directed DBE may be associated with better long-term outcomes because of the potential for fewer complications [36].

In conclusion, bowel preparation before capsule endoscopy is recommended for good visualization (therefore with higher diagnostic yield); the main indication for CE is obscure GI bleeding; in selected cases, CE can be useful for the other indications; large polypoid lesions may be missed by CE; diagnostic capabilities of CE and DBE are similar; CE is a good complemantary method for DBE.
